# Negligible transcriptome and metabolome alterations in RNAi insecticidal maize against *Monolepta hieroglyphica*

**DOI:** 10.1007/s00299-020-02582-4

**Published:** 2020-08-31

**Authors:** Xiaolei Zhang, Ruiying Zhang, Liang Li, Yang Yang, Yijia Ding, Haitao Guan, Xiaoqin Wang, Aihong Zhang, Hongtao Wen

**Affiliations:** 1 Quality and Safety Institute of Agricultural Products, Heilongjiang Academy of Agricultural Sciences, Harbin, 150086 China; 2 Biotechnology Research Institute, Chinese Academy of Agricultural Sciences, Beijing, 100081 China; 3 Beijing DaBeiNong Biotechnology Co., Ltd., Beijing, 100080 China

**Keywords:** Transcriptome, Metabolome, RNAi insecticidal maize, *Monolepta hieroglyphica* (motschulsky), Unintended effect

## Abstract

**Key message:**

**RNAi-based genetically modified maize resistant to Monolepta hieroglyphica (Motschulsky) was demonstrated with negligible transcriptome and metabolome alterations compared to its unmodified equivalent.**

**Abstract:**

As one of the most prevalent insect pests afflicting various crops, *Monolepta hieroglyphica* (Motschulsky) causes severe loss of agricultural and economic productivity for many years in China. In an effort to reduce damages, in this study, an RNA interference (RNAi)-based genetically modified (GM) maize was developed. It was engineered to produce *MhSnf7* double-stranded RNAs (dsRNAs), which can suppress the *Snf7* gene expression and then lead *M. hieroglyphica* to death*.* Field trail analysis confirmed the robustly insecticidal ability of the *MhSnf7* GM maize to resist damages by *M. hieroglyphica*. RNA sequencing analysis identified that only one gene was differentially expressed in the *MhSnf7* GM maize compared to non-GM maize, indicating that the transcriptome in *MhSnf7* GM maize is principally unaffected by the introduction of the *MhSnf7* dsRNA expression vector. Likewise, metabolomics analysis identified that only 8 out of 5787 metabolites were significantly changed. Hence, the integration of transcriptomics and metabolomics demonstrates that there are negligible differences between *MhSnf7* GM maize and its unmodified equivalent. This study not only presents a comprehensive assessment of cellular alteration in terms of gene transcription and metabolite abundance in RNAi-based GM maize, but also could be used as a reference for evaluating the unintended effect of GM crops.

**Electronic supplementary material:**

The online version of this article (10.1007/s00299-020-02582-4) contains supplementary material, which is available to authorized users.

## Introduction

As one of the major maize producers in the world, China has made outstanding contributions to global grain production. In 2018, the agricultural land dedicated to Chinese maize production increased to 42.13 million hectares. The total yield of maize was 257.17 million tons and accounted for 22.9% of global production (China Statistical Yearbook 2019). The rapid increase of Chinese maize production has promoted the stable development of the global agricultural economy. Currently, however, maize production has been significantly hampered by insect pests such as *Monolepta hieroglyphica.* As a member of the order Coleoptera, *M. hieroglyphica* breeds quickly and has gradually expanded its range in China since 2008 (Li et al. 2016; Liu et al. 2016). In addition to maize, *M. hieroglyphica* also damages soybean, peanut, and other crops. The broad host and geographic range coupled with high fecundity has positioned *M. hieroglyphica* as a major factor responsive for the drastic reduction in agricultural productivity with significant economic impact (Chen et al. 2016; Shi et al. 2017; Yan et al. 2019). At the present time, it is still a severe problem.

GM technology affords the most direct and effective way to combat a variety of bacterial, fungal, and insect pests that plague modern agriculture. For decades, GM crops such as maize (Wang et al. 2018), rice (Shen et al. 2019), tomato (Luan et al. 2016), cotton (Ni et al. 2017) and potato (Bagri et al. 2018) have been developed in response to a variety of agricultural problems and has resulted in crops with improved resistance to insects, disease, and environmental stress. GM crops, however, have been the subject of intense scrutiny regarding safety concerns especially in the context of public health (Schiemann et al. 2019). Although there has been no direct evidence to demonstrate any deleterious effects from GM crops on human health or the environment, it is essential that all GM safety issues are addressed including potential unintended effects prior to introducing GM crops or products for agricultural use (FAO/WHO 2000).

The so-called ‘‘Omics’’ technologies, including transcriptomics, proteomics and metabolomics, have been used not only for general biological analysis, but also more recently for safety assessment of transgenic plants (Zhou et al. 2012; Lambirth et al. 2015; Gayen et al. 2016; Rao et al. 2016; Van Emon 2016; Li et al. 2017; Abdullah et al. 2018; Ning et al. 2018; Peng et al. 2018; Wang et al. 2018). These methods allow for accurate detection of differentially expressed genes, proteins and metabolites relevant to a given plant phenotype as well as nutritional and toxicological characteristics (Harrigan and Chassy 2012). Besides, the analyses using these technologies are hypothetically stringent, which satisfy the unbiased assessment of any unintended effects (Rao et al. 2016). In this study, we utilized transcriptomics and metabolomics technologies to compare the cellular differentiation between RNAi-based GM maize and non-GM maize. Our data reveal that there is negligible differences between GM and non-GM plants, supporting the biosafety of the *MhSnf7* GM maize.

## Materials and methods

### Molecular cloning and genetic transformation

Total RNA from larvae of *M. hieroglyphica* was extracted by using Omega Plant RNA kit (Omega Bio-Tek, USA). Poly-oligo dT magnetic beads were used to extract mRNA from total RNA. cDNA was synthetized from the extracted mRNA by using Superscript II reverse transcritase kit (Invitrogen, USA). The primers were designed based on the *Snf7* gene sequence (Genbank ID XM_028287710.1) in National Center for Biotechnology Information (NCBI) database. The target fragments were amplified from the cDNA library with the following primer sets (Table 1) and a standard shuttle PCR protocol (95 °C for 3 min, 94 °C for 30 s and 55 °C for 30 s, 40 cycles). The target fragment, promoter and terminator were inserted into T-DNA to form expressing vector based on conventional molecular biological methods. The presence of the target fragment was confirmed by colony PCR after the vector was transformed into *Agrobacterium*.

**Table 1:** Primers used for target fragments cloning

Primer name	Primer sequence (5′–3′)
Snf 7-P1F	CTAGTCCTGGGGAGGCTATT
Snf 7-P1R	TCATCTTAGATGGGTGATTTC

Calli of *Zea mays* L. Zong 31 line were subjected to co-culture with *Agrobacterium* harboring plasmid. The transformation process followed a published procedure (Hiei et al. 1994). Regenerated *T*0 plants were grown in a growth chamber at a temperature of 24 ± 1 °C with 16 h light and 8 h dark at a relative humidity of 80%.

To detect the expression of dsRNA in plant, cDNA library was obtained from the leaves of GM maize *F*_2_ inbred line mRNA. The dsRNA was confirmed by specific PCR using the following primer sets (Table 2). The PCR reaction program was as follows: 95 °C for 3 min, 94 °C for 30 s and 55 °C for 30 s, 40 cycles.

**Table 2:** Primers used for dsRNA detection

Primer name	Primer sequence (5′–3′)
Snf 7-P3F	AGAGGAATACACCTTACTAGC
Snf 7-P3R	TGTAAGAGTTCCATCTATTTGC
qRT-CUL-F	GAAGAGCCGCAAAGTTATGG
qRT-CUL-R	ATGGTAGAAGTGGACGCACC

### Chamber trait analysis

The resistance of *MhSnf7* GM maize to *M. hieroglyphica* was evaluated using a bioassay method. Ten larvae of *M. hieroglyphica* (younger than 24 h) were added to each culture dish containing corn whorl leaves (3 leaf stage) and cultured in a climate-controlled chamber with the following conditions: 24 ± 2 °C, 70–80% relative humidity and 0 h day/24 h night photoperiod. Two days after inoculation, the proportion of living larvae was determined. Statistical analysis was performed using the Student’s *t* test.

### Transcriptome analysis

Four *MhSnf7* GM (5–6 leaf stage) and four non-GM maize plants (5–6 leaf stage) were randomly selected to serve as four biological replicates. Total RNA from maize leaves was extracted using Omega Plant RNA kit (Omega Bio-Tek, USA) and analyzed by agarose gel electrophoresis. Poly-oligo dT magnetic beads were used to extract mRNA from total RNA. The transcriptome sequence library was constructed using the NEBNext® Ultra™ RNA LibraryPrep Kit for Illumina® (NEB, USA) following the manufacturer’s instructions. The RNA-seq library was sequenced on the Illumina Hiseq 2000 platform. Clean reads were obtained by removing adapter sequences and omitting reads containing more than 10% unknown nucleotides or with low quality (containing more than 50% bases with *Q*-score ≤ 20).

Gene expression levels were evaluated in fragments per kilobase million (FPKM) based on the number of fragments mapped to the reference sequence. Differential expression analysis was performed using the DEGseq R package. Genes with an adjusted false discovery rate (FDR) < 0.05 and |log_2_ fold change|> 1 were scored as differentially expressed.

### UPLC–MS/MS metabolome analysis

Metabolites were extracted from 50 mg of *MhSnf7* GM and non-GM maize leaves from eight biological replicates using the solvent: 40% acetonitrile, 40% methanol, 20% water and 1 ng/μL 2-chloro-l-phenylalanine (internal standard). The samples were then vortexed for 30 s, homogenized at 45 Hz for 4 min, treated with ultrasound for 5 min in an ice-water bath, and stored − 20 °C for 1 h. The homogenate was then centrifuged at 13,500×*g* for 15 min at 4 °C. The resulting supernatants were transferred to vials and stored at − 80 °C until analysis.

UPLC–MS/MS analyses were performed using a UHPLC system (1290, Agilent Technologies) with a UPLC HSS T-3 column (2.1 × 100 mm, 1.8 μm) coupled to Q Exactive (Orbitrap MS, Thermo). The mobile phase A contained 0.1% formic acid and water. The mobile phase B was acetonitrile. The elution procedure parameters were as follows: 0 min, 1% B; 1 min, 1% B; 8 min, 99% B; 10 min, 99% B; 10.1 min, 1% B; 12 min, 1% B with a flow rate of 0.5 mL/min. The injection volume was 3 μL. For mass spectrometry, ESI source conditions were set as following: sheath gas flow rate as 45 Arb, aux gas flow rate as 15 Arb, capillary temperature 320 °C, full ms resolution as 70,000, MS/MS resolution as 17,500, collision energy as 20/40/60 eV in NCE model, spray voltage as 3.8 kV.

ProteoWizard software (version 3.0.4472) and R package XCMS (version 3.2) were used to conduct the MS raw data converting and processing. OSI-SMMS (version 1.0, Dalian ChemDataSolution Information Technology Co. Ltd.) was used for peak annotation. The data involving the sample name, peak number and normalized peak area were analyzed using SIMCA14.1 software package (V14.1, Sartorius Stedim Data Analytics AB, Umea, Sweden) for principal component analysis (PCA) and orthogonal projections to latent structures-discriminate analysis (OPLS-DA). Metabolites with variable importance in the projection (VIP) values exceeding 1 and a false discovery rate (FDR) < 0.05 were considered differentiated.

## Results

### Molecular characterization of *MhSnf7* GM maize

**Fig.1:**
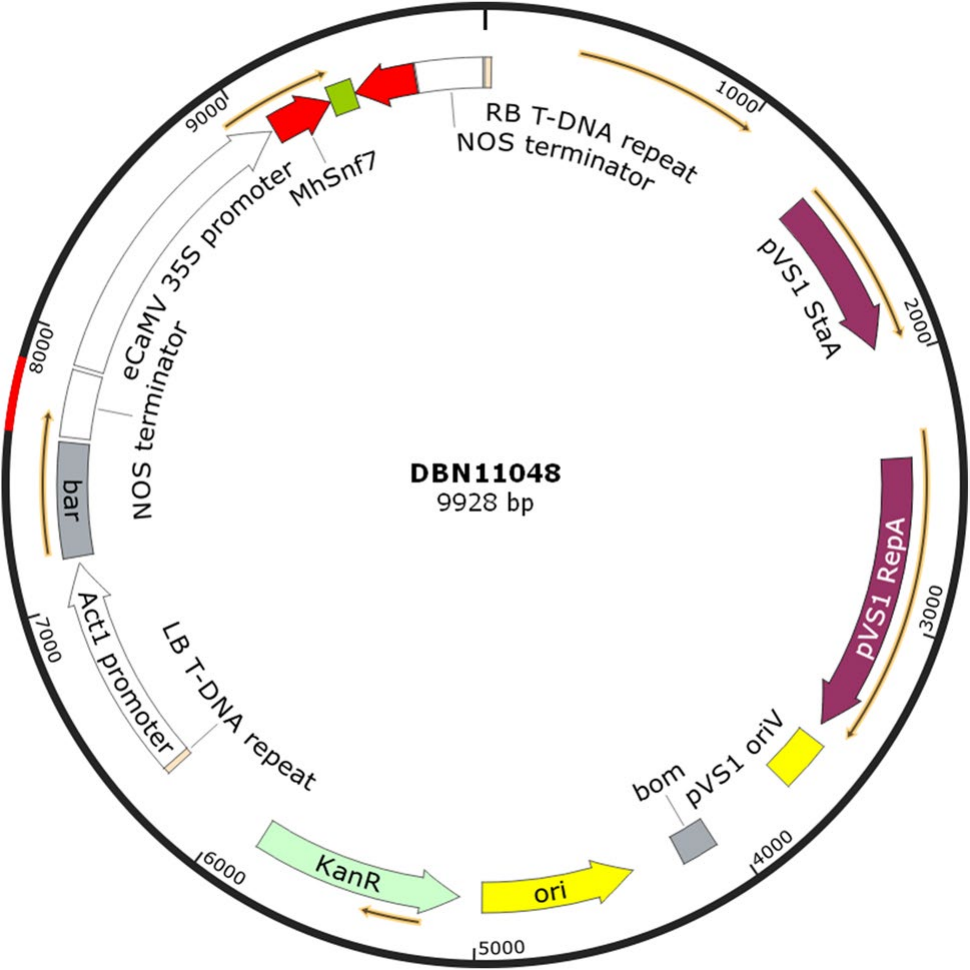
Schematic map of the *MhSnf7* dsRNA expression vector DBN11048. The vector contains eCaMV 35S promoter, which promotes the *MhSnf7* dsRNA with a high expression level. The selection marker is *bar* gene

The vector DBN11048 containing the target reverse complementary nucleotide sequence of *MhSnf7* was constructed as shown in Fig. 1. To assay for transgene integration after transformation, specific PCR was performed for simultaneous detection and the presence of stably integrated T-DNA in leaf genome was verified (Fig. S1). The third-generation sequencing technology detected that the vector was integrated into the tested maize 9 chromosome with single copy.

**Fig. 2:**
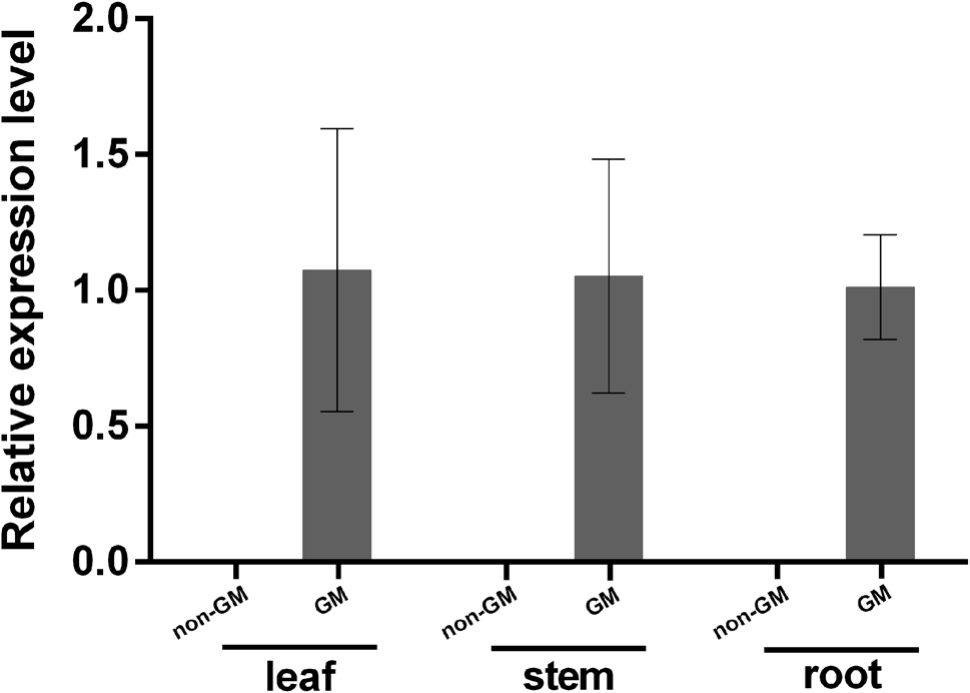
Relative expression analysis of *MhSnf7* dsRNA detected by qRT-PCR in the tissues of GM maize and non-GM maize. Mean ± SE was calculated for each tissue type. The values shown in this figure are the averages of three independent experiments. Error bars represent the SD (*n* = 3) of relative expression levels of *MhSnf7* dsRNA

The expression of the *MhSnf7* dsRNA in leaf, stem and root tissues was determined by fluorescence quantitative PCR. A dissociation curve showing a single peak at the melting temperature expected for that amplicon suggests specific amplification. The results showed that the *MhSnf7* dsRNA was highly expressed in all three tissues of GM maize, while there was no expression in the non-GM control (Fig. 2).

### Chamber trait analysis of MhSnf7 GM maize

**Fig. 3:**
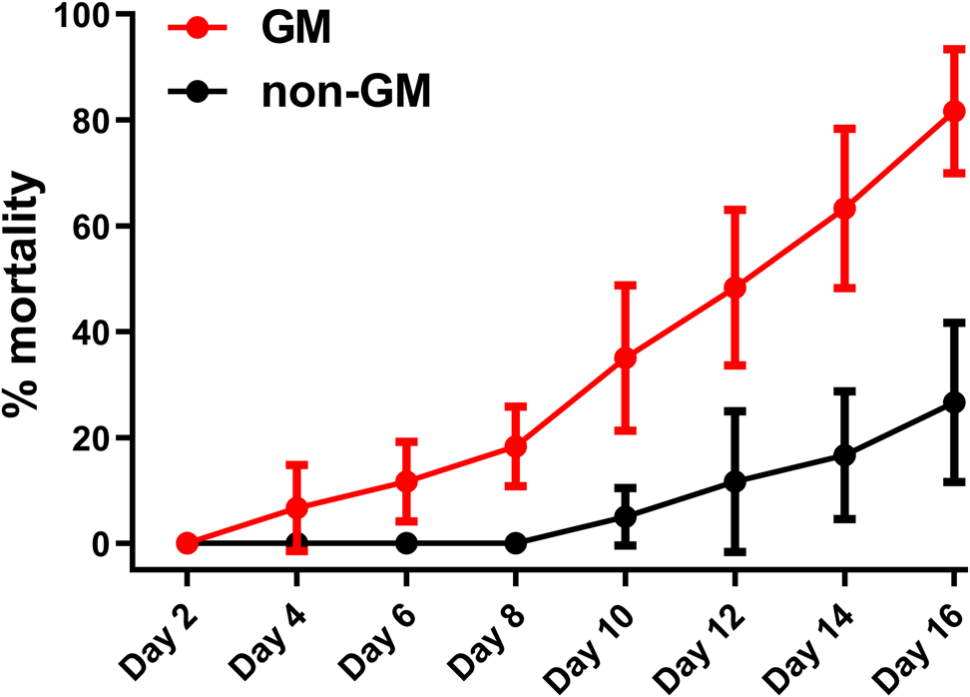
Insecticidal field trail analysis of *MhSnf7* GM maize. *M. hieroglyphica* larvae fed on GM maize and non-GM maize at time points as the x-axis. The mortalities as the y-axis are shown in red line and black line, respectively. Error bars represent the mortality SD (*n* = 10) of *M. hieroglyphica* larvae

Newly hatched larvae of *M. hieroglyphica* were fed with young leaves from *MhSnf7* GM and non-GM maize, respectively. After 16 days, the number of dead *M. hieroglyphica* larvae that had been fed on *MhSnf7* GM leaves was significantly greater compared to non-GM maize (*P* < 0.0001). The mortality rate (dead larvae/total larvae × 100%) on day 16 for the GM maize was approximately fourfold greater than that for non-GM maize (Fig. 3). This result demonstrates that the *MhSnf7* GM maize exhibits robust insecticidal capacity by demising *M. hieroglyphica* larvae.

### Transcriptome analysis of *MhSnf7* GM and non-GM maize

RNA-seq libraries prepared from *MhSnf7* GM and non-GM maize leaves were sequenced individually using the Illumina Hiseq 2000 platform. The high-quality clean reads from the two groups after raw read quality filtering were obtained (Table 3). Using the maize Ensembl release 34 AGPv4 as the reference genome, the average mapped ratios were 82.18% for *MhSnf7* GM group and 82.60% for non-GM maize group, respectively. Quality analysis showed that the two groups had an average of 55.61% and 55.65% GC (%) content of clean reads, 98.98% and 98.94% of Q20, and 96.71% and 96.61% of Q30, respectively. These data presented a high-quality sequencing, which can satisfy the subsequent analyses. Raw transcriptome sequences data have been deposited at NCBI Sequence Read Archive (SRA) under accession PRJNA546119.

**Table 3:** RNA-sequencing and quality analysis results

Sample	HQ clean reads number	Mapping ratio	Q20	Q30	GC
*T*-1	50077892	82.48%	98.96%	96.66%	55.32%
*T*-2	52977600	82.35%	98.95%	96.64%	55.44%
*T*-3	22649082	81.14%	99.01%	96.80%	55.76%
*T*-4	45179534	82.76%	98.98%	96.72%	55.74%
CK-1	47901628	82.33%	98.97%	96.69%	56.26%
CK-2	52459298	82.24%	98.93%	96.59%	55.82%
CK-3	55196970	82.95%	98.97%	96.67%	56.06%
CK-4	45438342	82.86%	98.88%	96.49%	54.46%

**Fig. 4:**
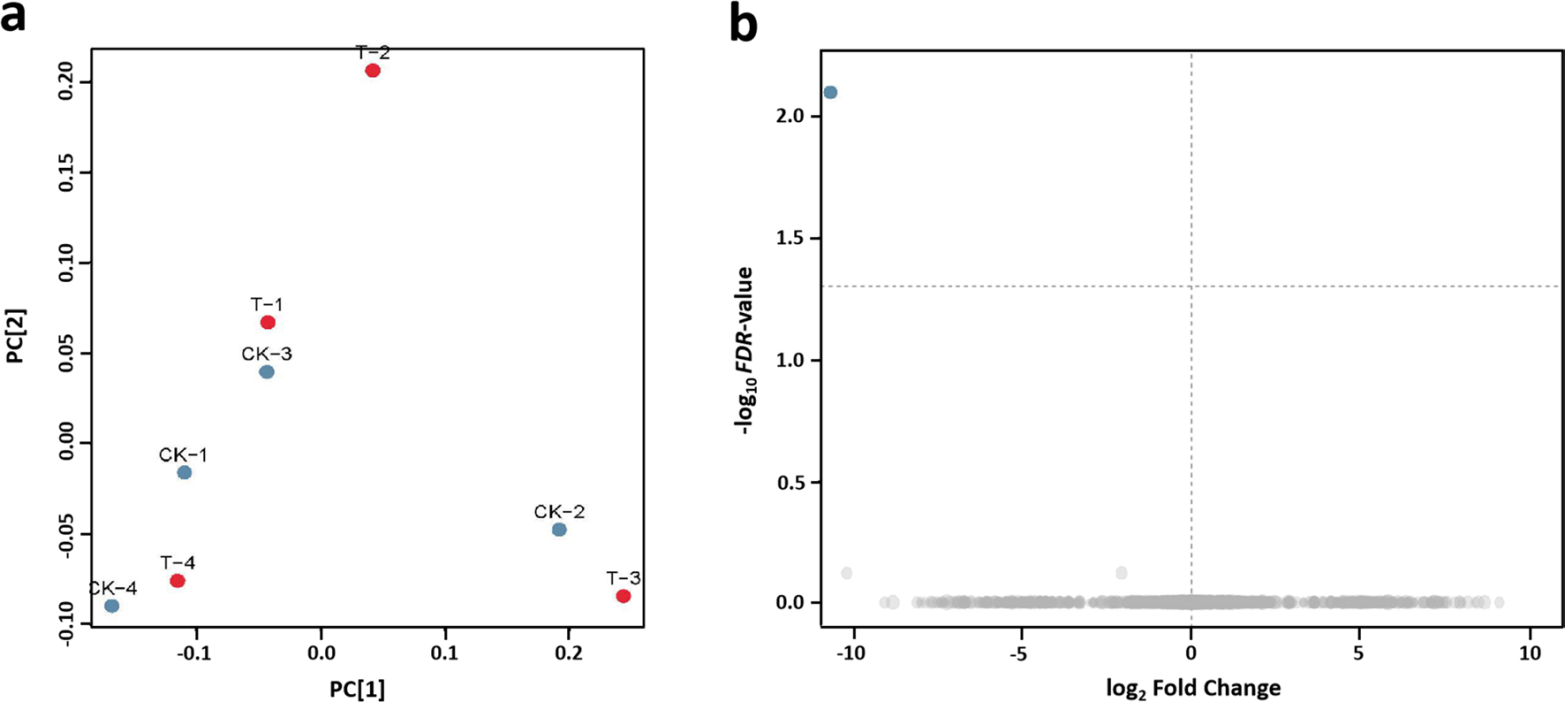
Differentially expressed genes analysis in GM and non-GM maize. **a** PCA analysis indicates the similarity of RNA-Seq datasets between the two groups. **b** Volcano plot with -log_10_ (*FDR*-value) from the *t* test as the y-axis and log_2_ (fold change) as the x-axis. The blue dot represents down-regulated gene with |log_2_ fold change|> 1 and FDR < 0.05, and the gray dots represent non-differentially expressed genes

An unsupervised model of PCA analysis was conducted, which can provide a natural and global informative look at the relationships between groups and would be used to formulate an initial biological conclusion (Worley and Powers 2013). The PCA analysis showed that the samples could not be separated, indicating that *MhSnf7* GM and non-GM maize groups were very similar at the transcriptional level (Fig. 4a). Under the threshold of FDR < 0.05 and |log_2_ fold change|> 1, we could identify that out of 31,635 detected genes, only one gene was differentially expressed (Fig. 4b). This result demonstrates that there is negligible transcriptome variation between the *MhSnf7* GM and non-GM maize.

### Metabolome analysis of MhSnf7 GM and non-GM maize

**Fig. 5:**
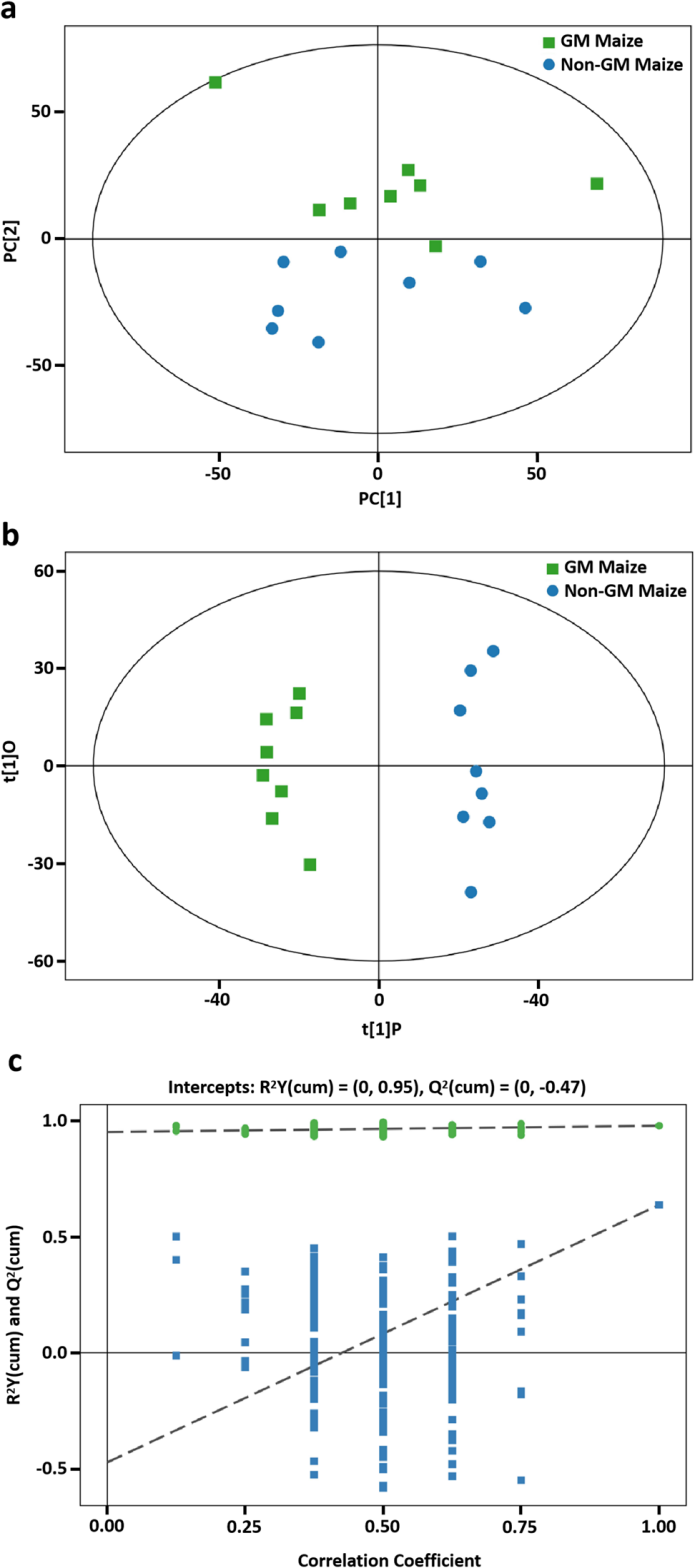
Differential metabolites analysis in GM and non-GM maize. **a** Score plot from PCA model. **b** Score plot from OPLS-DA model. **c** Corresponding validation plot from OPLS-DA model. Correlation coefficient as the x-axis represents the replacement reservation degree of replacement test, and the y-axis represents the value of *R*^2^*Y* (green dots) and *Q*^2^ (blue square dots). The two dashes represent the regression lines of *R*^2^*Y* and *Q*^2^, respectively

UPLC–MS/MS was performed on the metabolic extracts prepared from GM and non-GM maize leaves to address the changes in metabolome. PCA analysis showed that there was no marked trend of segregation between the two groups, indicative of the identical pattern of metabolome between *MhSnf7* GM and non-GM maize (Fig. 5a).

To maximize the discrimination between the two groups, we used OPLS-DA supervised multivariate data analysis to elucidate the different metabolic patterns. The score plot showed all samples were lying inside the 95% confidence interval (Hotelling’s *T*-squared ellipse) with clear separation and discrimination between the pairwise groups (Fig. 5b). The *R*^2^*Y* and *Q*^2^ intercept values of the permutation test were 0.95 and − 0.47, respectively (Fig. 5c). The *R*^2^*Y* close to 1 with low *Q*^2^ indicated that the established model had no overfitting phenomenon and can be used in subsequent analyses.

**Fig. 6:**
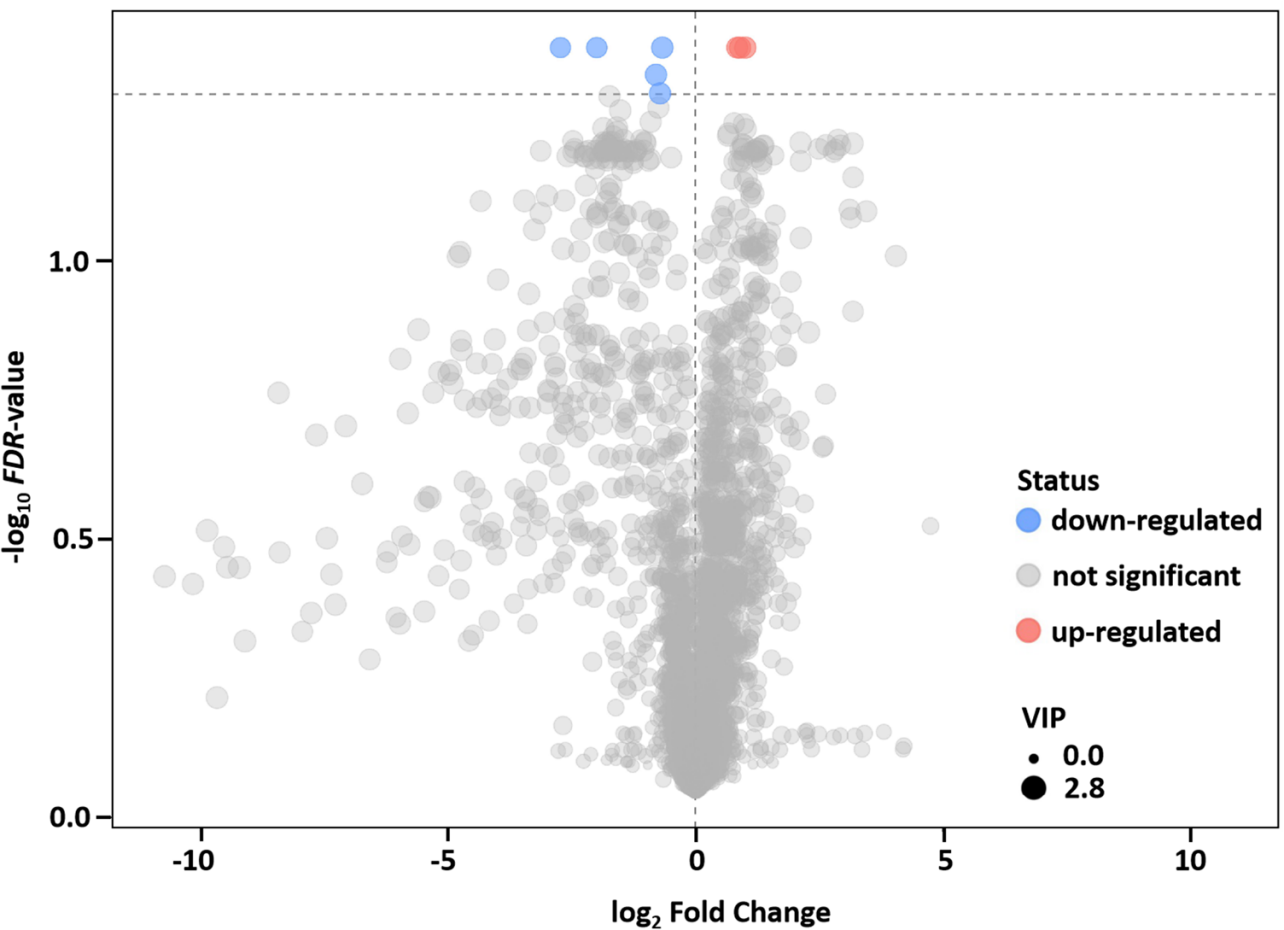
Volcano plot analysis for differential metabolite expression. log_2_ Fold change as the x-axis represents the multiple change of each metabolite and − log_10_ (FDR-value) from the *t* test as the y-axis. The significantly up-regulated metabolites are shown in red, while the significantly down-regulated metabolites are shown in blue, and the non-significantly different metabolites are shown in gray. The dots size represents the VIP value of the OPLS-DA model, and the larger the dot, the greater the VIP value

In total, 5787 metabolites were detected after preprocessing the raw data. The dataset of metabolome profiles included 172 compounds with known identity (Table S1). The metabolites identified as statistically significant were set with the cutoff of VIP > 1 and FDR < 0.05. Under this criteria, eight differential metabolites were determined between *MhSnf7* GM and non-GM maize (Table 4). Among these metabolites, five and three were down-regulated or up-regulated in GM maize relative to non-GM maize, respectively (Fig. 6).

**Table 4:** The differentially expressed metabolites between *MhSnf7* GM and non-GM maize

ID	MS2 name	MS2 score	VIP value	FDR value	log_2_ fold change
4949	Null	Null	2.1225	0.0412	− 2.7261
3710	Null	Null	2.1832	0.0412	− 1.9935
3628	Null	Null	2.4867	0.0461	− 0.7954
3266	Null	Null	2.4709	0.0498	− 0.7137
2002	Null	Null	2.5289	0.0412	− 0.6673
110	Gamma-Butyrolactone	0.5057	2.3865	0.0412	0.8481
2608	Null	Null	2.4935	0.0412	0.9032
4083	Null	Null	2.5038	0.0412	1.0089

## Discussion

Over the past decades, RNAi techniques have emerged as an eco-friendly, efficient, and reliable platform for the development of GM insecticidal plants (Mao et al. 2007; Bachman et al. 2013; Ni et al. 2017; Poreddy et al. 2017; Fu et al. 2019; Darsan Singh et al. 2019). These plants harbor double-stranded RNAs (dsRNAs) that can target and suppress mRNA levels through the RNAi pathway and lead to insect morbidity or mortality (Zhang et al. 2017). The interference sequence of exogenous genes inserted into plant genomic DNA can form a hairpin structure, which is recognized and degraded by Dicer after transcription (Vazquez et al. 2010). The inserted sequence will not be translated into protein. Hence, RNAi-based GM plants hold the attractive potential for not only improving crop quality and yield, but also avoiding the accumulation of exogenous proteins, and ultimately guaranteeing high biological safety. The first RNAi-based insect control trait expressing *DvSnf7* dsRNA was found to protect maize roots from feeding damage by the western corn rootworm (*Diabrotica virgifera virgifera*; WCR) (Baum et al. 2007; Bolognesi et al. 2012; Bachman et al. 2016). This discovery has enabled Monsanto Co. to successfully develop and commercialize the first RNAi insecticidal maize MON 87,411.

It is essential that GM plants should be evaluated and compared with their non-GM counterparts to ensure that the inserted sequence and the corresponding output do not negatively impact their food safety and nutritional equivalence (Ricroch et al. 2011). In this context, several studies have been conducted to investigate the unintended effect of GM plants using transcriptomics and metabolomics as evaluation criteria. Barros et al. (2010) identified that in glyphosate-tolerant transgenic maize, the alterations resulted from genetic modification in the levels of transcript, and protein and metabolite were fewer than those caused by environmental factors. Kim et al. (2013) found that the carotenoid-biofortified transgenic rice is substantially equivalent to its conventional counterpart at metabolic level. Rao et al. (2016) compared the metabolic changes in transgenic maize over-expressing the *Aspergillus niger phyA2* with its non-transgenic counterpart. They found that when natural variation was taken into consideration, the differential metabolites were only resulted from the target-engineered pathway in transgenic maize. Overall, all these former studies shed light on the biosafety of GM plants without unintended alteration in the cellular circumstance.

Although several researches have addressed the positive risk assessment of RNAi-based GM crops, the results are limited to ecological effects (Wolt et al. 2010; Bachman et al. 2016). To date, there is no report evaluating the safety of RNAi-based GM crops at the levels of transcriptome and metabolome. In this study, by integrating transcriptomics and metabolomics analysis, we revealed that there were only one gene and eight metabolites differentially expressed in *MhSnf7* GM maize. According to the report by Christ et al. (2017), transgenic BAR indeed converts plant endogenous aminoadipate and tryptophan to their respective N-acetylated products in several plant species. Our metabolomics data (Table S1) identified 172 compounds with known identity. However, the results did not cover the ectopic accumulation of acetyl-aminoadipate and acetyl-tryptophan from the non-specific *N*-acetyltransferase activities of transgenic BAR acting upon plant endogenous amino acids. These data offer unambiguous evidences that *MhSnf7* GM maize was almost identical to the non-GM equivalent. Therefore, our results expand current understanding of the transcriptome and metabolome changes in RNAi-based GM crops. We expect that this study could be used as a proper reference for evaluating the risk assessment of GM crops and ultimately benefit agricultural production in China.

## Electronic supplementary material

Below is the link to the electronic supplementary material.

Supplementary file1 (XLSX 757 kb)

Supplementary file2 (JPG 115 kb)
