# Supplementary figures and images for: Negligible transcriptome and metabolome alterations in RNAi insecticidal maize against Monolepta hieroglyphica
Source: Plant Cell Rep. 2020 Aug 31;39(11):1539–47. doi: 10.1007/s00299-020-02582-4 (PMC7554010; doi:10.1007/s00299-020-02582-4)

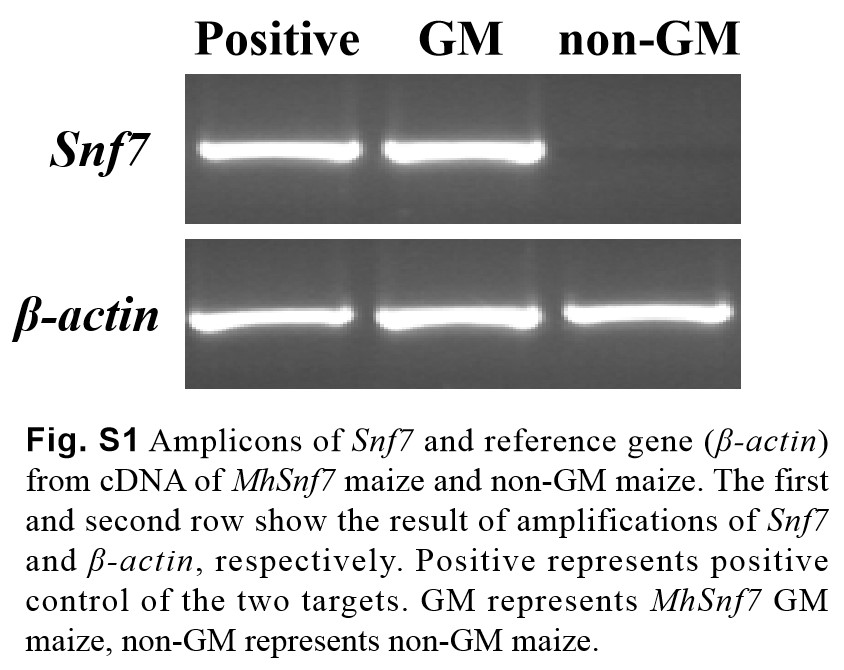

Supplement: Supplementary file 2 — Supplementary file2 (JPG 115 kb) [file 299_2020_2582_MOESM2_ESM.jpg]
